# A qualitative study on patients’ and their support persons’ preferences for receiving one longer consultation or two shorter consultations when being informed about allogeneic hematopoietic stem cell transplantation

**DOI:** 10.1186/s12913-021-06632-9

**Published:** 2021-06-30

**Authors:** Anne Herrmann, Ernst Holler, Matthias Edinger, Sascha Eickmann, Daniel Wolff

**Affiliations:** 1 Department of Epidemiology and Preventive Medicine, Medical Sociology, University of Regensburg, Regensburg, Germany; 2 School of Medicine and Public Health/University of Newcastle and Hunter Medical Research Institute, New Lambton Heights, Australia; 3 Department of Haematology and Internal Oncology, University Hospital Regensburg, Regensburg, Germany

**Keywords:** Hematological cancer, Stem cell transplantation, Patient preferences, Interviews, Qualitative research, Patient-centered care

## Abstract

**Background:**

Allogeneic hematopoietic stem cell transplantation (alloHSCT) is the only potentially curative treatment option for many patients with hematological disorders but it includes a significant risk of mortality and long-term morbidity. Many patients and their support persons feel overwhelmed when being informed about alloHSCT and may benefit from improvements in consultation style and timing.

**Aims:**

To explore, qualitatively, in a sample of hematological cancer patients and their support persons, their preferences for receiving one longer consultation or two shorter consultations when being informed about alloHSCT. Participants’ perceptions of when and how different consultation styles should be offered were also examined.

**Methods:**

Semi-structured face-to-face and phone interviews were conducted. A purposeful sampling frame was used. Data were analysed using framework analysis.

**Results:**

Twenty patients and 13 support persons were recruited (consent rate: 96%, response rate: 91%). Most patients (60%) and support persons (62%) preferred two shorter consultations over one longer consultation. This helped them digest and recall the information provided, remember questions they had, involve significant others and search for additional information. Patients would have liked to be offered paper and pen to take notes, take a break after 30 min and have their understanding checked at the end of the first consultation, e.g. using question prompt lists. Some patients and support persons preferred both consultations to happen on the same day to reduce waiting times as well as travel times and costs. Others preferred having a few days in-between both consultations to better help them prepare the second consultation. Participants reported varying preferences for different consultation styles depending on personal and disease-related characteristics, such as age, health literacy level and previous treatment.

**Conclusion:**

To our knowledge, this is the first qualitative study to explore patients’ and their support persons’ preferences for having one longer consultation or two shorter consultations when being informed about alloHSCT. Receiving two shorter consultations may help patients process and recall the information provided and more actively involve their support persons. Clinicians should consider offering patients and their support persons to take a break after 30 min, provide paper and pen as well as question prompt lists.

## Introduction

### Many patients struggle with understanding the information provided by their clinicians

When being presented with their diagnosis and treatment options, patients often have to digest and recall a plethora of information, and need to cope with the distress and anxiety related to their disease and treatment [1]. Patients are commonly introduced to abstract concepts, an unfamiliar language and a high degree of uncertainty regarding potential outcomes [1]. Many patients feel overwhelmed by the abundance of the information they receive and their emotions, which can interfere with their ability to recall and comprehend the information provided by their clinicians, leading to heightened anxiety, dissatisfaction with care, and inability to cope with illness [2, 3]. Support persons are one of the most important sources of information and advice for patients who often feel more certain about their care decisions after consulting their support persons [4, 5].

### Clinician-patient-communication on stem cell transplants is particularly challenging

Communication about diagnosis and treatment options can be particularly challenging for hematological cancer patients and their support persons, given the high symptom burden and frequently invasive and toxic treatment [6, 7]. Also, hematological cancers are often associated with high mortality and morbidity. Allogeneic hematopoietic stem cell transplantation (alloHSCT) is the only potentially curative treatment options for many patients but it can cause high physical and psychosocial stress, and includes a significant risk of mortality and long-term morbidity [8]. AlloHSCT can have debilitating long-term side effects patients and their support persons have to cope with. This includes Graft-versus-Host Disease (GvHD), an immunological disorder, caused by donor immune cells attacking recipients’ tissue which can affect almost every organ system [9]. GvHD remains a major cause of high rates of morbidity and mortality after alloHSCT and may include side-effects, such as nausea and loss of appetite, muscle weakness and cramps, osteoporosis, and an increased risk of bacterial, viral and fungal infections or future secondary malignancies [10, 11]. Thus, informing patients about the potential benefits and risks of alloHSCT can be challenging as it often involves delivering an abundance of complex, unfamiliar and distressing information to patients and their support persons.

### Differences in consultation styles may impact on patients’ understanding of information regarding their care

Cancer patients usually receive one longer consultation lasting approximately 40–60 min when being informed about their treatment [12, 13]. Clinical practice guidelines suggest that recall and understanding could be improved by providing two shorter, rather than one longer consultation [1, 14]. This may help patients and their support persons “digest” and use the information they were given. Studies indicate that cancer patients and their support persons may prefer this consultation style [15, 16]. However, findings are mixed as a number of patients wish to receive one longer rather than two shorter consultations. Also, providing one longer consultation remains common practice in many healthcare settings and patients may prefer to receive standard care [15, 16]. To our knowledge, there is no study exploring in-depth cancer patients’ and their support persons’ preferences for receiving two shorter or one longer consultation before making a treatment decision and providing *informed* consent. Qualitative research is particularly suited to fill this gap as it helps to provide in-depth insights into participants’ views and experiences and allows to investigate complex, but relatively unexplored areas of patient care [17]. A qualitative study on patients’ and support persons’ perceptions of receiving two shorter or one longer consultation may support the progression of research on this topic area and inform clinical practice about why and when to best offer which consultation style.

## Aims

This study explored, qualitatively, in a sample of hematological cancer patients prior to alloHSCT and their support persons, their preferences for receiving one longer or two shorter consultations when being informed about alloHSCT. Participants’ perceptions of when and how different consultation styles should be offered were also examined.

## Methods

### Design

Semi-structured interviews with hematological cancer patients and their support persons were conducted as part of a larger study which aimed to develop and test an intervention designed to improve clinician-patient-communication in the area of alloHSCT. Here we report on a nested descriptive study investigating participants’ communication experiences and preferences. A purposeful sampling frame was chosen to recruit participants from various socio-demographic and disease-related backgrounds. All methods were carried out in accordance with the Declaration of Helsinki and were approved by the ethics committee of the University of Regensburg (approval number: 19–136-101).

### Eligibility

*Patients* were eligible for this study if they i) had a confirmed diagnosis of hematological cancer, ii) were offered alloHSCT by their treating clinician, iii) were aged 18 years or older, iv) German speaking, and v) presenting for a consultation at the participating clinic. Patients who were judged by clinical staff to be physically or mentally incapable of completing the interview or who were unable to provide informed consent were excluded. *Support persons:* were eligible if they were nominated by the patient as someone helping them cope with their cancer through support, encouragement and communication. Support persons were aged 18 years or over, German speaking, and able to provide informed consent.

### Recruitment

Eligible patients were identified prior to their appointment by the transplant coordinator. The primary clinician taking care of the patient before transplantation informed the patient about the study and asked for consent to talk to a member of the research team who provided them with verbal and written study information, sought written informed consent and arranged a suitable time for a face-to-face or phone interview. Participants could choose the interview mode according to their availabilities and preferences. Consent was also sought from the support person accompanying the patient to their appointment. If they were not present, the patient was asked to pass a recruitment package onto their support person. The age and gender of non-consenting patient-support person dyads was recorded to examine consent bias.

### Data collection

The interviewer encouraged patients and support persons to tell their story about the information they have received regarding their diagnosis and treatment options and how treatment decisions were made, in the way they preferred, with as little interruption as possible from the interviewer. This narrative approach aimed to elicit the variety and interplay of factors related to diagnosis and treatment discussions. The initial narrative was followed by semi-structured open-ended questions asking participants about optimal information provision, psychological concerns and strategies to improve the quality of care. This included questions on whether and why patients and support persons would prefer to receive one longer or two shorter consultations. At the end of the interview, participants were given the option to provide additional comments. The questions were informed by a review of the literature and discussions amongst the research team. Data collection was stopped when data saturation was perceived to be reached and further data gathering was not likely to reveal additional findings to answer the research question [18, 19].

### Data analysis

Interviews were transcribed verbatim. Transcripts were checked for accuracy by one researcher (AH) and analyzed using framework analysis. This approach belongs to a broad family of qualitative data analysis methods often related to as “thematic analysis” or “qualitative content analysis” [20]. Framework analysis provides a systematic model for mapping and interpreting qualitative data and was thus considered appropriate to develop a profound in-depth understanding of participants’ communication experiences and preferences. Both descriptive and explanatory conclusions were drawn from the data. Each interview served as unit of analysis [21]. Initially, all interviews were coded openly by applying a paraphrase (i.e. a “code”) that described what had been interpreted in the passage as important. This inductive approach aimed to minimize bias and to ensure all relevant codes were captured. Codes were then grouped around more complex categories by summarizing and synthesizing the range and diversity of coded data in order to form an analytical framework [20, 22]. The framework was discussed with another member of the research team (DW) and applied to subsequent interviews. If a new code or category emerged, the framework was revised accordingly to ensure all data were captured, regardless of whether they fitted in the existing framework. This helped us validate and extend conceptually the underlying framework. The robustness of the developed hypotheses was tested on the basis of each single case as well as independently of individuals and thus beyond single cases [23]. Threads of meaning (“themes”) were then generated across categories by interpreting and explaining the categories and establishing associations between them. All conclusions drawn from the data were reviewed and discussed by all members of the research team. Demographics were analyzed using appropriate summary statistics. Chi-squared tests were used to assess consent bias for age and gender.

## Results

### Sample characteristics

Participants were recruited between April and September 2019. Recruitment yielded a consent rate of 96% and a response rate of 91%. A median of 14.5 days elapsed between the consultation and the interview. Interviews lasted on average 36 min. Patients had a mean age of 56 years (SD: 11). Most were female and diagnosed with leukemia. Support persons had a mean age of 48 years (SD: 20, see Table 1). The majority of support persons were the patient’s partner and living with the patient (see Table 2). There were no statistically significant differences between consenters and non-consenters in terms of age or gender (*p* > 0.05). The following themes emerged from the data: (i) feeling of being overwhelmed as a reason for preferring two shorter consultations over one longer consultation; (ii) perception of being well informed and increased travel times and costs as reasons for preferring one longer consultation; (iii) discordance of patients’ and support persons’ views; (iv) perceptions of when and how to provide two shorter rather than one longer consultation.

**Table 1:** Sociodemographic and disease-related characteristics of patients

Characteristic	Patients ***n*** = 20 (%)
**Age mean (SD)**	56 (11)
**Gender**	
Male	7 (35)
Female	13 (65)
**Diagnosis**	
Leukemia	9 (45)
Lymphoma	2 (10)
Multiple Myeloma	3 (15)
Myelofibrosis	3 (15)
Other	3 (15)
**Citizenship**	
German	18 (90)
Other	2 (10)
**Highest level of education**	
Vocational training	17 (85)
University degree	2 (10)
None	1 (5)

**Table 2:** Sociodemographic characteristics of support persons

Characteristic	Support persons ***n*** = 13 (%)
**Age mean (SD)**	48 (20)
**Gender**	
Male	7 (54)
Female	6 (46)
**Relationship to patient**	
Partner	10 (77)
Relative	2 (15)
Friend	1 (8)
**Living with patient**	
Yes	9 (69)
No	4 (31)
**Highest level of education**	
Vocational training	12 (92)
University degree	1 (8)

### Themes

#### Feeling of being overwhelmed as a reason for preferring two shorter consultations over one longer consultation

Patients commonly received one longer consultation with their treating physician. They were offered written information at the end of their consultation and were given the opportunity to contact their treatment team at any time should they have further questions. Most patients (60%, *n* = 12) and support persons (62%, *n* = 8) would have preferred to receive two shorter consultations rather than one longer consultation since they felt overwhelmed by the information given by their treating clinicians. Most patients reported reaching a point where no further information intake was possible due to the sheer abundance of details provided and the technical terms used. They described that *“the sponge was full at some point. ( …*)” and required time to *“(m) ake room again for new thoughts. Or just clear thoughts.” (patient, female, 45 y, acute myeloid leukemia).* Others wished for two shorter consultations with a break in-between to give them time to:

*(…) vent the brain a little (…) Because then you have room to maneuver again. You are receptive again. Because after an hour, yes, that’s the case everywhere. Then you are in a room and you know that CO2 makes you tired anyway. (…) A break, that would be ideal. (patient, female, 61 y, multiple myeloma)*

Many patients reported pondering on details their clinicians had mentioned although the conservation had already moved on to the next topic, resulting in patients losing track of what their clinician was explaining to them. Most interviewees indicated signing the consent forms for the recommended treatment without having fully understood the potential benefits, risks and modes of the treatment. Few patients also felt intimated by the long waiting time prior to their consultation or the academic titles of their treating clinician. This feeling of being intimidated increased patients’ distress and their difficulties to recall the information they were given.

*I was very nervous and had respect and a bit of fear sitting in front of such a professor, I must honestly say. The demigods in white. And I was very tense. I absorbed 75% and then at the end I forgot 25%, they were not as registered as they should have been. That was also the time when we had 38 degrees. You’re already sucked out like an earthworm (…) then it can happen that you wait an hour for him. (…) (=Having two shorter consultations) that would be a great thing. (…) always two, three, four topics (…) that information is enough. You can at least process that (…). (patient, male, 54 y, acute myeloid leukemia).*

*The doctor says: “I still need your signature here so that we can start the treatment.” (…) My partner just signed it. At that moment you just sign it and say okay I trust you. If it said we would go on our next holiday to Hawaii then we would have signed it, honestly. (…) (Y)ou don’t really read the thing (=the consent form) until a day or two later. Then you ask yourself: What did I actually sign now and what exactly is going on? (support person, partner, male, 37 y).*

*Sometimes he (=the treating clinician) just came with examples, where you think about how they work. And then you just stay there, and then you realize again that you have to stay in conversation. But from time to time you are side-tracked. (patient, male, 65y, primary myelofibrosis).*

Most patients reported that they did not have any further questions when being asked by their clinician at the end of the consultation. Many of these patients wished for a break in-between two consultations to allow them to consider what has been said, take notes and raise further questions during the second consultation. Time in-between two consultations also allowed patients and their support persons to look at additional information, such as the brochure provided by their clinician or further information they found online. Patients appreciated having the opportunity to digest what they heard during the consultation, maybe even in the comfort of their own homes and then start searching for additional information they required to understand what the clinician had told them. They felt that this could also help them gather questions to be raised in the second consultation.

*There are the technical terms, which are actually too much for me. But when I looked something up on the Internet, what it was, I really understood. (patient, female, 65 y, acute myeloid leukemia).*

*Yes, I got these brochures, leukemia booklets and I read them. But at home. I was quite interested in them. But at the end of the conversation you’re done. Because you have to digest it first. (patient, female, 71 y, acute myeloid leukemia).*

A number of patients felt very distressed while being informed about alloHSCT and its potential side-effects. These patients indicated that having a break in-between two shorter consultations would have helped them to relax a bit, deal with their anxiety and distress and gather their thoughts. Many appreciated the opportunity to drink something in-between two conversations, to refresh both body and mind. Attending two shorter consultations rather than one longer consultation also allowed patients to more actively involve their support persons, by enabling them to share with their support person what they have understood and identify further questions they would like to raise during the second consultation. A number of support persons shared this view and reported that they would have appreciated a break that would have allowed them to better process and recall the information provided.

*There is a lot of information that you get in a short time. And of course this information is not exactly positive. (…) It’s extreme. You think again and again if all goes well. (support person, partner, male 36 y).*

*Because after the conversation we talked again as a family. Everyone brought the information they remembered back to the table. And of course, questions arise again. In other words, a longer break in the middle would be very good for me personally. (support person, daughter, 27 y).*

*The informal exchange in between (=the consultations) where you do a little something to process the whole thing a little, I think it’s important. Because then it has actually already settled down a bit and then you can follow-up. So I would welcome that. That’s good. (patient, female, 61 y, multiple myeloma).*

*I didn’t know what to expect (=from the consultation) and if I had known I would have loved to take my wife with me. (…) A “neutral person” has a completely different question and a different need for information in areas that are not so relevant to me. When I got home, I already knew what I should have asked. (patient, male, 68 y, primary myelofibrosis).*

#### Perception of being well informed and increased travel times and costs as reasons for preferring one longer consultation

A number of patients (40%, *n* = 8,) preferred one longer over two shorter consultations as they felt well informed and got their questions answered either during the first consultation and/or by searching for further information elsewhere (e.g. online). Others felt that the myriad of information provided by their clinicians was so overwhelming that they would have struggled raising their questions independent of the length of the consultation. Some patients also wanted to be given all information straight away rather than having to wait for further details to be provided during the second consultation. Most patients and support persons reported having to travel long distances to the clinic which would make attending a second appointment difficult due to increased travel times and costs.

*I want to hear everything right away. And then, if there is no question, then I make my break feeling reassured. Then I want to take a deep breath. (…) We had a long way from X (=hometown) to the clinic. We wanted to come home as soon as possible. Therefore, we listened to the doctor from A to the last letter, everything he said and then we drove home. So without a break it was better. (support person, partner, male, 62 y).*

*When you walk out the door, of course you think back: Why didn’t I ask that? But if it had been a quarter of an hour and we had gone out, then I would probably have stood at the door and asked the same question. (patient, male, 68 y, primary myelofibrosis).*

Some patients indicated that the consultation was interrupted by frequent phone calls their treating clinician had to answer leaving them enough time to digest the information given to them. Others felt reassured by the fact that their treatment team offered them to call at any time should they have further questions. This made a second consultation redundant for them. Patients and support persons also pointed out that preferences for consultation style and timing may be dependent on the characteristics of each individual patient, including age or health literacy level, but also on situational or disease-related factors, such as whether the consultation took place soon after diagnosis or previous treatment. Patients and support persons explained that older patients or those who have just received their diagnosis may struggle with understanding and digesting information on alloHSCT, making it more likely that these patients would benefit from having two shorter consultations rather than one longer one.

*I think it depends a lot on the individual personalities (…) If my mother had been there for example, I think after an hour she would have said: What has happened now? (…) But it’s also situation-dependent, it’s very difficult with such a diagnosis no matter what age. (…) At that moment you’re completely taken by surprise, you’re completely exhausted by the whole topic of what to expect. (support persons, partner, male, 37 y).*

*I think it’s better if it goes all at once and then it’s over. And then the secretary sitting in the anteroom also said that we can call them any time if we have questions. And she was also very nice. And she asked again and again if everything was all right. (patient, female 35 y, acute lymphoblastic leukemia).*

*It (=the consultation) was interrupted by calls and then I didn’t have the feeling that I was overwhelmed by a topic. (patient, male, 68 y, acute myeloid leukemia).*

#### Discordance of patients’ and support persons’ views

Some patients-support persons-dyads differed in their preferences for two shorter or one longer consultation. If there was discordance, support persons preferred two shorter consultations over one longer one, whereas the patients they cared for wished for one longer consultation. Many support persons felt that this was because patients were more distressed than them, since they were the ones having to cope with treatment and its side-effects. They thought that patients would still benefit from receiving two shorter consultations but preferred one longer one to avoid further potentially distressing information. Some support persons were not present during all consultations the patients they cared for had with their treating clinicians. They often reported difficulties with understanding all relevant information and preferred two over one consultation to help them understand and process all important details. Others were unsure about how to ask all questions they had during the consultation, particularly if they related to the patient’s prognosis. This was because support persons did not want to upset the patient by receiving further emotionally charged information. These support persons preferred two shorter consultations over one longer one as this would allow them to think about potentially upsetting questions and chat to the patient they cared for about when and how to best ask them.

*I am often reluctant to ask the last question, if she (=the patient) sits beside me, because I think I don’t like that now at all, that she hears all that. (support person, friend, female, 53 y) … Yes, there are some things that I consciously don’t hear. (…) It is probably such a protective mechanism, you don’t want to hear that then. (patient, 53 y, female, multiple myeloma).*

*I was a bit slain by what was coming to X (=the patient). (support person, sister, 46 y) …. Because you didn’t get the other conversations with the other doctors. (patient, 64 y, male, acute myeloid leukemia) … Yes, I didn’t get that, that’s true. (support person, sister, 46 y).*

#### Perceptions of when and how to provide two shorter rather than one longer consultation

Patients and support persons who had to travel long distances to the clinic but still preferred to receive two shorter over one longer consultation wished for the second consultation to take place on the same day as the first consultation. This was to reduce travel times and costs associated with their clinic visits. Some patients indicated that a break in-between two consultations could be used to provide further care, such as taking blood or talking to a patient navigator about what to expect post-transplant. However, views differed: A number of patients wished for the break in-between two consultations to be free of other “duties”, to allow for some spare time to relax and gather their thoughts. They highlighted that the room where patients and support persons spend their break in-between two consultations should convey a neutral atmosphere and allow for the consumption of beverages.

*So maybe not directly in the consultation room but you sit down in a room where the atmosphere is a bit more “comfortable”. There you can have a water and a coffee for example. You just loosen it up a bit. (patient, female, 29 y, acute lymphoblastic leukemia).*

*You can just sit in the cafeteria for half an hour, three quarters of an hour. And then eat a snack, drink coffee, talk about it and then it goes on again. (support persons, partner, female, 56 y).*

*And the blood sample with the break did not bother me at all. Meanwhile we went for a cup of coffee. (patient, female, 45 y, acute myeloid leukemia).*

*But the whole thing (=the consultation) interrupted with lung test function, that gets you a bit off track. Then you are mentally somewhere else. (patient, female, 61 y, multiple myeloma).*

Although a number of patients wished for both consultations to occur on the same day, some interviewees indicated that they preferred the second consultation to take place a few days after the first consultation. They felt that this would allow them sufficient time to digest the information provided by their clinicians and to chat to their support persons. Few patients also reported that the side-effects of their previous treatment, such as chemotherapy, would affect their ability to focus and recall information making it easier for them to attend the second consultation a few days after the first one.

*If, for example, it’s chemo, it’s a bit better after a week or two (…) but on the same day, then just an hour or two later, that doesn’t help at all. (…) because when I’m knackered, I’m knackered. For me then the whole day is gone. (patient, male, 49 y, B-cell lymphoma).*

*You have to leave it (=the information provided by the clinician) lying around for a while, definitely a few days. (patient, female, 45 y, acute myeloid leukemia).*

Patients indicated that it would be helpful if clinicians offered patients and their support persons paper and pen during the first consultation. This would allow and encourage them to take notes of things they found important and/or wanted to know more about. Some patients indicated that it may be helpful if clinicians checked patients’ understanding after the first consultation to get a better sense of where to start at the second consultation and ensure patients remembered all important details. Patients also reported that such recall checks could occur with the help of written questionnaires or question prompt lists patients and their support persons could take home to consider during the break in-between two consultations.

*The clinician said at the beginning that we would go through our questions at the end and there was just a lot of information. Perhaps it would have been better, more pleasant for me, to say that you might subdivide it (=the consultation) (…), then take a break and say: “Did you understand everything there?” So you were forced to remember that for later. (patient, female, 58 y, prolymphocytic leukemia).*

*Maybe you let the patient fill out something afterwards, whether they understood it at all, that you do things like a questionnaire about the conversation. (…) Then you have something in writing, you can take it home with you, then you can process it differently if you can read it again in peace. I would suggest that something like that happens. (patient, male, 54 y, acute myeloid leukemia).*

*If we had a small block and a pencil which can be used by several patients that would be good, so that the budget (=of the clinic) is not overstrained. (patient, male, 68 y, primary myelofibrosis).*

Some patients indicated that the break in-between two consultations could occur after 30 min. Others suggested a flexible approach where clinicians would ask patients during the first consultation whether they still feel able to take in further information and schedule the break when needed. Patients also highlighted that it would be important to ensure that the second consultation does not cause significant additional waiting times. However, many patients noted that these suggestions may be hard to realize given shortages of staff and time pressure on clinicians.

*Maybe half an hour or a little more would do (…) For example the two of us (=patient and wife), we talk a lot with each other. That you can do that a little bit more. (…) And then build on that again and carry on. (patient, female, 61 y, multiple myeloma).*

*You could make it very flexible, so you don’t say you make blocks of hours or half blocks of hours, but just in the course of the conversation maybe you just ask to see whether you (=the patient) are still receptive and then decide should we take a break. I think that would make sense. (support persons, partner, male 37 y).*

*But I don’t think it works that way (=having two shorter consultations and a break in-between) because then a patient comes who has to go somewhere else and then you sit there for hours waiting for the second part of the conversation. (patient, female, 62 y, primary myelofibrosis).*

## Discussion

To our knowledge, this is the first qualitative study to provide in-depth insights into cancer patients’ and their support persons’ preferences for receiving one longer or two shorter consultations when being informed about alloHSCT [15, 16]. Most participants in our study preferred to receive two shorter consultations over one longer consultation. This allowed them to better digest and recall the information provided by their clinicians, involve their support persons and search for additional information to increase their understanding of what their clinician had told them.

AlloHSCT is one of the most widespread and accepted treatment options for many patients suffering from hematological malignancies [24]. However, it also involves a myriad of risks and side-effects patients have to understand and recall [25]. Thus, informing patients about alloHSCT and making a treatment decision in line with patients’ wishes and needs can be challenging [26]. Research in this area indicates that patients often feel not well informed about alloHSCT [27]. Additionally, research showed misperceptions of risks and benefits of the treatment with cancer patients overestimating their prognosis, which could lead to depression and anxiety during treatment cycles and a decrease in overall quality of life [27, 28]. This is often due to patients and support persons not being fully informed about their prognosis and treatment options before they even see their transplant specialist. To improve care in this area and enhance patient outcomes information provision by the referring physicians could be refined. Some transplant centres offer additional written information for their patients to consider at home after the consultation and provide the opportunity to contact the treatment team, which often involves psycho-oncologists, should patients or support persons have further questions. Such strategies may help patients and support persons increase understanding and recall of the information provided by their clinicians [1, 29]. Providing two shorter rather than one longer consultation may be another way to provide high-quality patient-centred care. It may also be a strategy that could be applied to other areas of oncology where clinicians need to deliver a myriad of complex information to their patients. Future research should explore whether our findings could be applied to other settings and patient populations.

Patients indicated that a break after 30 min may be sensible and that they would find it helpful to be offered paper and pen to take notes during the consultations with their clinicians. Writing down keywords might aid patients’ thought structure and thus facilitate a second consultation. Previous experimental studies have been conducted with video and tape recording, which showed a significant boost in remembering parts of the consultation and higher patient involvement during follow-up [30]. This is in accordance with the so-called Ebbinghaus curve, which assumes that 90% of information is forgotten which is only heard or seen once. Retaining information becomes much easier if the information can be obtained multiple times [31]. Providing patients and their support persons with paper and pen to take notes might thus also be of benefit when conveying information on the risks and side-effects of alloHSCT.

Many patients and their support persons in our study wished that their clinicians would ask them at the end of the first consultation about what they had understood and provided written check lists or question prompt lists to help them comprehend and consider the information provided. They also felt that this would help them prepare questions to be raised in the second consultation. A number of studies have suggested that question prompt lists can improve patient outcomes, by helping patients become more engaged in the consultation and at the same time enabling them to meet their information needs. There also seems to be a greater involvement of the accompanying support person [32–36]. Other strategies to increase recall may be sharing of clinical notes for example via an electronic patient record or open notes, or even translating them into lay language [37]. Such strategies may be acceptable to patients, support persons and clinicians and may provide a deeper patient understanding of the risks and benefits of their diagnosis, prognosis and the treatments available to them, which could lead in turn to a better adherence and thus improved health outcomes [38, 39]. However, more research is required on the effectiveness of such strategies and potential barriers and enablers to their implementation into clinical practice.

Having a break in-between two shorter consultations may also be an opportunity to provide further patient information, such as patient decision aids. Decision aids are designed to help patients decide on their care by presenting unbiased information on the options available to them [40, 41]. They have been developed in different formats, such as written booklets or online information [42, 43]. A recent Cochrane review concluded that compared to usual care decision aids help patients feel better informed, clearer about their values, have more accurate risk perceptions and become more involved in decision making across a wide variety of decision contexts [44]. However, little is known about how to implement written patient information, such as decision aids, into clinical practice [45, 46]. Our results indicate that patients and their support persons may prefer to receive written patient information during the first consultation with their clinicians to consider before their next appointment. Future intervention studies should test whether being provided with written patient information to consider in-between two consultations, rather than during one longer consultation, can improve patient outcomes.

Our results are in line with previous studies suggesting that patients’ and their support persons’ information preferences differ according to personal and disease-related characteristics, such as age, health literacy level or time since diagnosis [47–50]. Situational factors, such as distances between patients’ homes and the clinic they attended, also influenced their preferences for different consultation styles. Some patients and support persons in our study wished for both consultations to happen on the same day to reduce waiting times as well as time and costs associated with travelling to and from the clinic. Others preferred having a few days in-between two consultations to help them process all information and prepare the second consultation. Asking patients about their preferences may be the first step to ensure that care is tailored to their individual needs and wishes [51, 52]. However, such an approach may be difficult to implement into the daily routines of busy clinics [53]. Given the time pressure many clinicians suffer from and long waiting times for patients, clinicians may not always be able to provide the consultation style each individual patient would prefer. It may be worth considering whether waiting and travel times could be minimized by using video consultations, so that patients, support persons and clinicians benefit. Telemedicine has already gained its place in other medical specialties such as rural general practice [54–56]. During the Covid-19 pandemic, the field of telemedicine received a new impulse [57, 58]. Recent studies suggest that the use of telemedicine may be acceptable to patients and clinicians in settings where it had not been considered before, such as psychiatry and oncology/haematology, if participants also have the opportunity to see their treating specialist in person [59, 60]. Thus, it may be worthwhile considering providing the first consultation face-to-face and the second one using telemedicine. Preliminary data indicate positive impacts of the use of telemedicine on important outcomes, such as improved patient satisfaction with the consultation, decreased travel times and costs for patients and clinicians and increase of timely referrals [61]. Well informed patients are also more likely to adhere to their prescribed care and report a great degree of trust in and satisfaction with their clinicians [62, 63]. This may ultimately save time later on in patients’ care trajectory as greater patient understanding may allow for more succinct discussion later on, decrease patients’ level of anxiety and lower the number of follow-up appointments needed [64–66].

### Limitations

Preferences for how information should be provided may change dependent on where patients are in their care trajectory [67, 68]. Given that we recruited participants at one point in time we could not confidently assess such dynamics of change. Future longitudinal studies should investigate this issue. Also, our results are not intended to be statistically representative, nor to be representative of other healthcare settings and services. However, they provide important in-depth insights into participants views and preferences and add to the quantitative data in this area [15, 16]. This study was focused on patients’ and support persons perceptions related to alloHSCT. Patients and support persons facing other treatment modalities may have different views. However, alloHSCT presents a complex treatment regime requiring patients and their support persons to understand a myriad of information. It is thus well suited to explore patients’ and support persons’ perceptions of different consultations styles to help them process this information. Also, we explored participants’ perceptions and what they thought how different consultation styles would impact on their outcomes. Future intervention studies should assess the effectiveness of the proposed consultation styles in improving patient and support person outcomes. Given that not all patients had support persons willing to participate in this study, we could not recruit an equal number of patients and support persons. We conducted face-to-face interviews in the participating clinic and phone interviews. It has been suggested that phone interviews may provide less rich data than face-to-face interviews but evidence to support this assumption is lacking [69]. Also, participants in our study could choose the interview mode to reduce research-related burden and many appreciated the opportunity to conduct the interview on the phone in the comfort of their homes. We interviewed patients and support persons together. This is in line with previous studies emphasizing that interviewing individuals and their supportive others together could generate richer data than interviewing them separately due to the interaction between the interviewees leading to a more fully presentation of the studied phenomena [70–72]. Finally, we do not know how clinicians’ communication skills may have impacted on participants’ preferences since we did not observe the consultations.

## Conclusions

To our knowledge, this is the first qualitative study to explore in-depth patients’ and their support persons’ preferences for one longer consultation or two shorter consultations when being informed about alloHSCT. Our data suggest that having two shorter rather than one longer consultation may help patients and their support persons comprehend the information provided by their clinicians, think of questions they would like to have answered, search for additional information and become more actively involved in decisions regarding their care. Clinicians should consider offering patients and their support persons paper and pen to take notes, take a break after 30 min of consultation and provide check lists for patients’ understanding as well as question prompt lists to consider in-between two consultations. Given the differences in patients’ and support persons’ preferences and in an attempt to provide optimal, patient-centred care, clinicians should ask patients about their preferences for different consultation styles.

## Data Availability

The dataset generated and analysed during the current study is not publicly available due to identification of the interview partners but is available from the corresponding author on reasonable request.
